# Impact of Genetic Diversity and Genome Plasticity of *Leishmania* spp. in Treatment and the Search for Novel Chemotherapeutic Targets

**DOI:** 10.3389/fcimb.2022.826287

**Published:** 2022-01-24

**Authors:** Ana Maria Murta Santi, Silvane Maria Fonseca Murta

**Affiliations:** Grupo de Genômica Funcional de Parasitos – Instituto René Rachou, Fundação Oswaldo Cruz, Belo Horizonte, Brazil

**Keywords:** *Leishmania*, chemotherapy, drug resistance, genetic diversity, genome plasticity

## Abstract

Leishmaniasis is one of the major public health concerns in Latin America, Africa, Asia, and Europe. The absence of vaccines for human use and the lack of effective vector control programs make chemotherapy the main strategy to control all forms of the disease. However, the high toxicity of available drugs, limited choice of therapeutic agents, and occurrence of drug-resistant parasite strains are the main challenges related to chemotherapy. Currently, only a small number of drugs are available for leishmaniasis treatment, including pentavalent antimonials (Sb^V^), amphotericin B and its formulations, miltefosine, paromomycin sulphate, and pentamidine isethionate. In addition to drug toxicity, therapeutic failure of leishmaniasis is a serious concern. The occurrence of drug-resistant parasites is one of the causes of therapeutic failure and is closely related to the diversity of parasites in this genus. Owing to the enormous plasticity of the genome, resistance can occur by altering different metabolic pathways, demonstrating that resistance mechanisms are multifactorial and extremely complex. Genetic variability and genome plasticity cause not only the available drugs to have limitations, but also make the search for new drugs challenging. Here, we examined the biological characteristics of parasites that hinder drug discovery.

## Introduction

Leishmaniases are a complex of diseases caused by different species of protozoan parasites of the genus *Leishmania*, which are transmitted to humans by the bite of infected female sand fly insects. Leishmaniases are found in 90 countries and territories (WHO, 2021) and are a major public health concern because they have high death rates among all neglected diseases, and they mostly affect the poorest populations (Kaufer et al., 2017; Parthasarathy and Kalesh, 2020).

**Figure 1:**
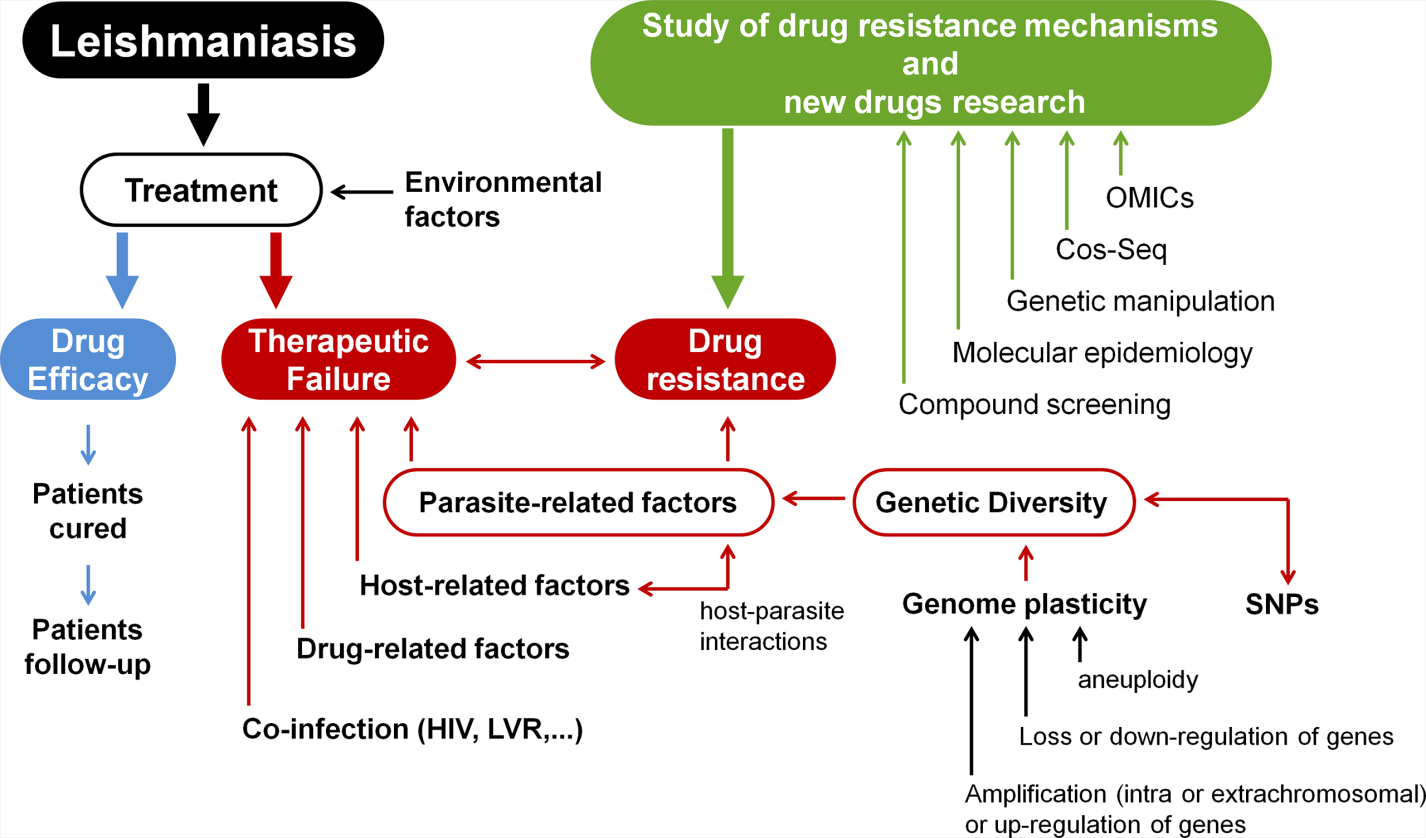
Research into resistance mechanisms and the development of new drugs for leishmaniases treatment. Environmental variables, parasite-related factors, host-related factors, drug-related factors, and co-infections (HIV, LVR) can all affect how well a patient responds to therapy. This demonstrates the significance of studying host-*Leishmania* interactions in the context of developing novel chemotherapeutic agents for leishmaniasis. The parasite’s diversity is another key element in treatment outcomes since diversity is closely related to drug resistance. Because *Leishmania*’s genome is highly plastic, there is a great deal of variety across samples. Different *Leishmania* isolates display single-nucleotide polymorphisms (SNPs), as well as structural variations (such as changes in the numbers of genes, clusters of genes, or even whole chromosomes). Thus, genome plasticity involves different molecular modifications, such as amplification or up-regulation of genes, loss or down-regulation of genes, or aneuploidy. These modifications contribute to drug-resistant phenotypes in *Leishmania* samples. Consequently, drug resistance mechanisms are multifactorial and exceedingly complicated. Different approaches can be used to study resistance mechanisms in *Leishmania* and also to search for new molecular targets and novel drugs. OMICs integration (such as genomics, transcriptomics, proteomics, and metabolomics) can be used to better understand pathways associated with drug metabolism. However, due to post-transcriptional control, genomic and transcriptomic data should be interpreted with caution. In addition, data from promastigotes should be carefully analysed since such amastigote forms have distinct transcriptomic and proteomic profiles. The high-throughput Cos-Seq method can be used to identify gain-of-function resistance mechanisms and drug targets. Genetic manipulation of parasites can be performed using different molecular tools (such as deletion by allelic substitution, overexpression and heterologous expression, plasmid shuffling, RNAi, DiCRE, DD domain, Cos-Seq method, and CRISPR/Cas9) which allow the identification of novel chemotherapeutic targets. Molecular epidemiology is also important to better understand the diversity of the genus *Leishmania* and the variables that drive diversification. Finally, compound screening is a critical component in the search for novel drugs (Modified from Ponte-Sucre et al., 2017).

Currently, no vaccine for human use is available to prevent leishmaniasis. Controlling reservoirs and vectors is advised; however, this is extremely complicated to accomplish. Thus, rapid diagnosis and treatment of patients is the main form of disease control (WHO, 2021). Only a small number of compounds are used to treat leishmaniasis, including pentavalent antimonials (Sb^V^), amphotericin B and its formulations, miltefosine, paromomycin sulphate, and pentamidine isethionate (Muraca et al., 2020). Therapeutic failure in leishmaniasis is a growing problem and may be related to the occurrence of treatment-resistant parasites, but also to other factors, such as patient immunity (Lopez-Velez et al., 1998; Alvar et al., 2008; Van Griensven et al., 2014); nutritional status, age, and gender of the patient (Dorlo et al., 2012; Ostyn et al., 2014); and whether the parasites are infected with any RNA viruses, such as LRV1 (Bourreau et al., 2015; Adaui et al., 2016). Here, we provide an overview of how genetic variation in the genus *Leishmania* influences the emergence of drug-resistant parasites, as well as the main tools for studying drug resistance mechanisms and searching for new drugs. The topics covered in this study are summarised in **Figure 1**.

## The Genome of *Leishmania* Is Atypical

Different species of *Leishmania* have different numbers of chromosomes as well as diverse sets of genes (Ivens et al., 2005; Peacock et al., 2007). A recent assembly of the genome of *L. major* revealed a 32.8 Mb genome involving 11,238 genes distributed across 36 chromosomes (Camacho et al., 2021). Previously, *Leishmania* populations were thought to have an average diploid genome, but mosaic aneuploidy is now thought to be the norm in the genomes of these parasites, with the degree of aneuploidy varying depending on species or strain (Sterkers et al., 2012; Lachaud et al., 2014).

Almost all protein-coding genes in trypanosomatids do not contain introns and are organised in unidirectional polycistronic transcription units with no functionally linked genes. Pre-mRNAs generated by polycistronic transcription are processed to produce mature mRNAs (Bartholomeu et al., 2021). All genes are constitutively expressed, mainly by RNA polymerase II; however, the start of transcription is not well understood because canonical promoter sequences have not yet been found in these parasites (Martínez-Calvillo et al., 2003; Martínez-Calvillo et al., 2004; Thomas et al., 2009; Iantorno et al., 2017). Epigenetic mechanisms appear to play a role in *Leishmania* transcription initiation by influencing DNA accessibility (Thomas et al., 2009; Chandra et al., 2017; Saha, 2020; Grünebast et al., 2021). Transcription termination occurs at the end of each polycistronic transcription unit and is determined by the presence of the base J (van Luenen et al., 2012; Reynolds et al., 2016; Kieft et al., 2020). However, it is commonly stated in the literature that in the absence of transcriptional regulation, the regulation of protein expression in these parasites is mediated by post-transcriptional events, such as RNA degradation, translation control, and protein degradation (Clayton and Shapira, 2007; Grünebast and Clos, 2020; Karamysheva et al., 2020). Several studies have shown that chromosomal copy number can be associated with transcription levels, supporting the notion that expression control occurs after transcription (Dumetz et al., 2017; Iantorno et al., 2017; Patino et al., 2019). In contrast, transcript and protein levels are not necessarily correlated (Alcolea et al., 2019).

## Importance of Genetic Diversity and Genome Plasticity of *Leishmania*

Variations in the parasite genome can be associated with its geographical distribution and clinical manifestations, which can influence leishmaniasis management. Interestingly, large-scale research involving a large number of samples and wide geographic range has revealed that genetic diversity is significantly higher than that previously reported (Bussotti et al., 2018; Zackay et al., 2018; Franssen et al., 2020; Imamura et al., 2020; Patino et al., 2020; Salloum et al., 2020; Zheng et al., 2020; Patino et al., 2021; Schwabl et al., 2021). Recently, single-cell sequencing has demonstrated the presence of several distinct karyotypes within the same *Leishmania* clone (Imamura et al., 2020; Negreira et al., 2020), and multiple-genotype infections have been demonstrated to occur even within the same host and tissue (Cupolillo et al., 2020).

In addition to the role of mutations in parasite diversity (Downing et al., 2011; Domagalska et al., 2019; Franssen et al., 2020), the *Leishmania* genome is highly plastic and constantly rearranges, resulting in variations in gene copy number, clusters of genes, or even whole chromosomes (Ubeda et al., 2008; Leprohon et al., 2009; Mannaert et al., 2012; Laffitte et al., 2016b; Iantorno et al., 2017). As a result, mosaic aneuploidy is not only widespread in *Leishmania* but is also an essential adaptive mechanism that allows a certain genome structure to be quickly selected in the face of adverse conditions (Sterkers et al., 2012; Dujardin et al., 2014; Lachaud et al., 2014; Sterkers et al., 2014; Reis-Cunha et al., 2018). Alterations in ploidy are not random, but seem to follow the same pattern in samples subjected to different stressors, and each strain tends to follow the same pattern, which indicates the occurrence of selective processes (Dumetz et al., 2017; Bussotti et al., 2018; Restrepo et al., 2019).

The copy number of a gene can be changed by adding or deleting genes in tandem, or by creating extrachromosomal copies of genes, which can be linear or circular. These extrachromosomal gene copies are commonly found in *Leishmania* under stress, but are also found in wild-type populations (Ouellette and Borst, 1991; Grondin et al., 1993; Leprohon et al., 2009). It has been reported that there are pairs of repeated sequences surrounding sets of genes in *Leishmania* (Leprohon et al., 2009; Ubeda et al., 2014; Bussotti et al., 2018; Carnielli et al., 2018) and DNA double-strand breaks near or within these repeated sequences may induce homologous recombination, which is associated with an increase in gene rearrangements (Genois et al., 2014; da Silva, 2021).

Subtelomeric DNA is more sensitive to replicative stress (Damasceno et al., 2013; Damasceno et al., 2016; Damasceno et al., 2018), and telomeric amplification has also been identified as a genetic adaptability mechanism in *Leishmania* (Bussotti et al., 2018). Interestingly, aneuploidy may arise from the duplication of subtelomeres outside the S phase (Damasceno et al., 2020a).

*Leishmania* replicates by clonal expansion; however, several studies have reported genetic exchanges between parasites, suggesting that *Leishmania* may reproduce sexually during the life cycle (Gutiérrez-Corbo et al., 2021). It has been proposed that this exchange may be important for the long-term survival of these parasites (Van den Broeck et al., 2020; Kato et al., 2021).

## Genomic Diversity Among Drug-Resistant Parasite Strains

Drug resistance is not an unexpected finding in these parasites, and it has been proposed that genetic variation is the primary driving force in the emergence of diverse drug-resistant phenotypes (Decuypere et al., 2012; Reis-Cunha et al., 2018). Because a variety of alterations might result in resistance to currently available treatments, there is no unique marker to evaluate resistance in clinical isolates. Here, we focus on certain examples where genetic diversity led to resistance in both clinical isolates and in *in vitro* studies.

### Antimony

Antimonials include meglumine antimoniate (Glucantime^®^) and sodium stibogluconate (Pentostam^®^). Several studies have shown that clinical isolates have increased resistance to pentavalent antimony (Peters, 1981; Sundar, 1994; Romero et al., 2001; Sundar, 2001; Croft et al., 2006; Azeredo-Coutinho et al., 2007; Perry et al., 2015; Mohebali et al., 2019). These clinical isolates have high variability in terms of antimonial resistance mechanisms, and the same sample can present several different alterations. For example, it was demonstrated that *L. braziliensis* and *L. panamensis* resistant to trivalent antimony (Sb^III^) exhibit differences in chromosomal somy and gene copy number when compared to their respective susceptible lines; however, such changes are more prominent in *L. braziliensis* (Patino et al., 2019). Different antimony-resistance mechanisms have been reported, including decreased cellular antimony entry, decreased drug reduction/activation, increased antimony efflux, and sequestration of the metal-thiol conjugate into intracellular vesicles of *Leishmania* (Croft et al., 2006).

An intriguing example of how genetic diversity might aid in the development of resistant parasite strains is how high levels of As (III) in Indian waters may have assisted in the selection of antimony-resistant parasites (Perry et al., 2015). Several different mechanisms can be involved in the Sb^III^ resistant phenotype in India, as it was shown, for example, that *L. donovani* from India has pre-adaptative aneuploidies involving various chromosomes (Dumetz et al., 2018). As *MRPA* (previously known as *PGPA*) is an ATP-binding cassette gene implicated in Sb^III^ sequestration into intracellular vesicles (Leprohon et al., 2009), it has been suggested that amplification of the *MRPA* gene copy number could generate the Sb^III^ or As^III^ resistance phenotype in samples, because both have the same sequestration mechanism (Maciaszczyk-Dziubinska et al., 2012; Dumetz et al., 2018). Similarly, other studies have shown that aneuploidy is related to the acquisition of antimony resistance in *Leishmania* by altering the *MRPA* copy number (Haimeur et al., 2000; Légaré et al., 2001; Anacleto et al., 2003; Mukherjee et al., 2007; Leprohon et al., 2009; Moreira et al., 2013).

However, other mechanisms can also participate in parasite resistance to Sb^III^, such as changes in the aquaglyceroporin (*AQP1*) coding sequence or down-regulation of expression of this gene in clinical samples (Mandal et al., 2010; Dumetz et al., 2018; Potvin et al., 2021) because it is known that differences in AQP1 transporter levels can influence both Sb^III^ and As^III^ uptake (Gourbal et al., 2004; Marquis et al., 2005; Mandal et al., 2010). On the other hand, amplification of trypanothione synthetase (*TryS*) may also play a role in resistance because an increase in trypanothione levels favours the formation of conjugates with Sb^III^ or As^III^, which increases its sequestration into intracellular vesicles (Mukhopadhyay et al., 1996; Frézard et al., 2014; Dumetz et al., 2018).

Mutations can also generate resistance against Sb^III^; for example, mutations in the multidrug resistance 1 gene (*MDR1*) have been associated with drug resistance in samples from individuals who do not respond to treatment (Abadi et al., 2021), while calcium-dependent protein kinase (*CDPK1*) mutations are linked to resistance to both paromomycin and antimony (Bhattacharya et al., 2019).

Another useful technique for assessing potential resistance pathways is to obtain resistant parasites *in vitro*. It is worth noting that the detected genes are frequently similar to those found in clinical samples. *In vitro* Sb^III^ resistance selection results in parasites with amplification of many genes, including *MRPA*, ascorbate-dependent peroxidase (*APX*), and a putative glucose-6-phosphate dehydrogenase (*G6PDH*) (Leprohon et al., 2009; Mukherjee et al., 2013; Monte-Neto et al., 2015). Obtaining SbIII-resistant parasites *in vitro* can also result in deletions or point mutations in a region containing *AQP1* (Mukherjee et al., 2013; Monte-Neto et al., 2015).

Furthermore, overexpression of antioxidant defence enzymes, such as iron superoxide dismutase-A (Tessarollo et al., 2015), tryparedoxin peroxidase (Andrade and Murta, 2014), or APX (Moreira et al., 2018) are involved in the Sb^III^-resistant phenotype in *L. braziliensis*.

### Miltefosine

Miltefosine is the only oral medicine available for the treatment of leishmaniasis and was first used in India to replace antimonials (Sundar et al., 2000; Sundar et al., 2002). Despite the usefulness of this drug in treating diseases caused by some species of *Leishmania*, the limited efficacy of miltefosine in treating visceral leishmaniasis in Brazil has been related to the loss of the Miltefosine Sensitivity Locus (*MSL*) (De Morais-Teixeira and Damasceno, 2011; Carnielli et al., 2018; Carnielli et al., 2019). Mutations in the miltefosine transporter confer resistance to both miltefosine and amphotericin B (Coelho et al., 2012; Fernandez-Prada et al., 2016; Laffitte et al., 2016a). In contrast, it was recently demonstrated that increasing the number of copies of the *Ros3* (*Lem3p/CDC50*) gene in clinical isolates of *L. braziliensis* increases miltefosine uptake, rendering these parasites more sensitive to treatment (Espada et al., 2021).

### Amphotericin B

Analysis of clinical isolates of *L. donovani* resistant to amphotericin B revealed a greater rate of amphotericin B efflux due to increased expression of the multidrug resistance gene *MDR1* (Purkait et al., 2012). Studies have also shown that amphotericin B-resistant parasites can be easily selected for *in vitro* (Mbongo et al., 1998; Al-Mohammed et al., 2005). Loss of the gene encoding 24-sterol methyltransferase (*SMT*) has also been associated with *L. donovani in vitro* resistance to amphotericin B (Rastrojo et al., 2018). Similarly, another study selected four *L. mexicana* lines with amphotericin B resistance induced *in vitro*, one of which showed a resistance-associated mutation in the sterol biosynthesis gene sterol C5-desaturase (*SC5D*), and three lines revealed loss of expression of SMT due to genomic copy number variants (Pountain et al., 2019). Amphotericin B resistance has also been reported in *L. mexicana* owing to mutations in the sequence of sterol 14-demethylase (*CYP51*) (Mwenechanya et al., 2017).

### Drug Combinations

Combinations of drugs to treat leishmaniasis, in which different drugs inhibit different metabolic pathways, seem to represent a promising option for overcoming resistance in parasites that are as adaptable as *Leishmania*. Drug combinations can reduce the overall dose of drugs required and duration of treatment, and can lead to lower toxicity and improved patient compliance (Uliana et al., 2018). However, drug combinations for leishmaniasis treatment must be used with caution as resistance to multiple drugs and cross-resistance can occur (García-Hernández et al., 2012; Berg et al., 2015; Fernandez-Prada et al., 2016).

## Impact of Genetic Diversity on New Drug Research

The search for new molecular targets for leishmaniasis treatment is an urgent task. Compound screening is an important approach for identifying new drugs. However, because of the tremendous diversity of these parasites, it is exceedingly difficult to identify drugs that are effective in treating infections caused by different species or strains. In this sense, many “-omics” (such as genomics, transcriptomics, proteomics, and metabolomics) have demonstrated diversity among the *Leishmania* genus and how various pathways play important roles in the resistant phenotype. In addition, the use of “-omics” to study host-*Leishmania* interactions should not be underestimated because such interactions are also important in terms of treatment outcomes. However, as *Leishmania* exhibits post-transcriptional control, there is a risk of extrapolating genomic or transcriptomic data in the context of drug resistance. Sample preparation, in contrast, is the key concealing element in proteome and metabolomic analysis. Furthermore, data from promastigotes should be interpreted with caution, because amastigote forms have distinct transcriptomic and proteomic profiles (De Pablos et al., 2016). Other “-omics” obstacles, such as operating costs, complexity, and the broad range of samples, will most likely be addressed in the next years as “-omics” and bioinformatic technologies progress (Dos Santos et al., 2016).

In addition to the evaluation of “-omics”, gene manipulation in parasites is an interesting option to better understand the pathways associated with drug metabolism. Currently, several tools can be used for genetic manipulation of *Leishmania*, such as the classic method of deletion by allelic substitution (Cruz et al., 1991), overexpression and heterologous expression (Kapler et al., 1990), plasmid shuffling (Murta et al., 2009), RNAi (Lye et al., 2010), DiCRE (Duncan et al., 2016), DD domain (Madeira et al., 2009), and CRISPR/Cas9 (Sollelis et al., 2015; Zhang and Matlashewski, 2015). In particular, the LeishGEdit toolkit has allowed researchers to explore the function of hundreds of genes swiftly and effectively, propelling the research of gene function and regulation of *Leishmania* metabolic pathways to unprecedented levels (Beneke et al., 2017; Beneke and Gluenz, 2019; Beneke and Gluenz, 2020). Another novel approach is the high-throughput Cos-Seq method, which can be used to identify gain-of-function resistance mechanisms and drug targets (Gazanion et al., 2016; Fernandez-Prada et al., 2018). In addition to these tools, sequencing of the parasite genome is also fundamental (Ivens et al., 2005), and the availability of parasite genomes in the TritrypDB database provides extremely easy access to all relevant data (Aslett et al., 2010).

However, despite all of the tools available, conducting studies involving gene manipulation in these parasites is not always straightforward, mainly because of the remarkable plasticity of their genomes and its high sensitivity to environmental changes. The parasites can undergo several adaptations for survival *in vitro*, and it has been recommended that genetic manipulations should be conducted directly in clinical samples to avoid experimental artefacts (Dumetz et al., 2017). Similarly, gene deletion can lead to the selection of parasites that display aneuploidies and altered phenotypes that may not match those observed in nature (Santi et al., 2021). Unfortunately, inducible systems to turn gene expression on and off in *Leishmania* have not been explored, and there is limited literature regarding the use of these systems (Yan et al., 2001; Kushnir et al., 2005; Yao et al., 2007; Kraeva et al., 2014). Although the implementation of the DiCRE system offers significant advancement in this regard, there are still certain limitations because it is neither reversible nor tunable (Duncan et al., 2016; Santos et al., 2017; Damasceno et al., 2020b; Damianou et al., 2020).

## Conclusions and Future Directions

Here, we emphasised the relevance of research into the diversity of the genus *Leishmania* and the variables that drive diversification. We reaffirm the relevance of using different approaches, such as “-omics” technologies, genetic manipulation, and compound screening, to elucidate drug resistance mechanisms and identify novel chemotherapeutic targets for leishmaniasis. Single-cell sequencing will reveal many more varieties previously submerged under the parasite pool. We further emphasise the need for molecular techniques to explore pathway regulation and establish novel inducible systems for *Leishmania*. An integrated study using data provided by these different approaches and aspects of host-*Leishmania* interactions will contribute to a better understanding of the complexity of drug resistance mechanisms, therapeutic failure, and great adaptability of these insidious parasites.

## Author Contributions

SM and AS designed the work, collected data, wrote and revised the manuscript. All authors contributed to the article and approved the submitted version.

## Funding

This work received financial support from the following agencies: Programa INOVA FIOCRUZ - Fundação Oswaldo Cruz (VPPCB-007-FIO-18-2-94); Convênio Fiocruz-Institut Pasteur-USP (no grant number); Fundação de Amparo à Pesquisa do Estado de Minas Gerais (FAPEMIG – APQ-02816-21), Conselho Nacional de Desenvolvimento Científico e Tecnológico (CNPq 304158/2019-4), and Coordenação de Aperfeiçoamento de Pessoal de Nível Superior - Brasil (CAPES) - Finance Code 001. SM is supported by CNPq. A.M.M. Santi is supported by CAPES.

## Conflict of Interest

The authors declare that the research was conducted in the absence of any commercial or financial relationships that could be construed as a potential conflict of interest. 

## Publisher’s Note

All claims expressed in this article are solely those of the authors and do not necessarily represent those of their affiliated organizations, or those of the publisher, the editors and the reviewers. Any product that may be evaluated in this article, or claim that may be made by its manufacturer, is not guaranteed or endorsed by the publisher.
